# LegumeGRN: A Gene Regulatory Network Prediction Server for Functional and Comparative Studies

**DOI:** 10.1371/journal.pone.0067434

**Published:** 2013-07-03

**Authors:** Mingyi Wang, Jerome Verdier, Vagner A. Benedito, Yuhong Tang, Jeremy D. Murray, Yinbing Ge, Jörg D. Becker, Helena Carvalho, Christian Rogers, Michael Udvardi, Ji He

**Affiliations:** 1 Division of Plant Biology, The Samuel Roberts Noble Foundation, Ardmore, Oklahoma, United States of America; 2 Division of Plant & Soil Sciences, West Virginia University, Morgantown, West Virginia, United States of America; 3 Department of Cell and Developmental Biology, John Innes Centre, Colney, Norwich, United Kingdom; 4 Plant Genomics Lab, Instituto Gulbenkian de Ciência, Oeiras, Portugal; 5 Institute for Molecular and Cell Biology, University of Porto, Porto, Portugal; 6 Cancer Genomics Research Laboratory, Division of Cancer Epidemiology and Genetics, National Cancer Institute, SAIC-Frederick, Inc., Bethesda, Maryland, United States of America; National Institute of Genomic Medicine, Mexico

## Abstract

Building accurate gene regulatory networks (GRNs) from high-throughput gene expression data is a long-standing challenge. However, with the emergence of new algorithms combined with the increase of transcriptomic data availability, it is now reachable. To help biologists to investigate gene regulatory relationships, we developed a web-based computational service to build, analyze and visualize GRNs that govern various biological processes. The web server is preloaded with all available Affymetrix GeneChip-based transcriptomic and annotation data from the three model legume species, i.e., *Medicago truncatula*, *Lotus japonicus* and *Glycine max*. Users can also upload their own transcriptomic and transcription factor datasets from any other species/organisms to analyze their in-house experiments. Users are able to select which experiments, genes and algorithms they will consider to perform their GRN analysis. To achieve this flexibility and improve prediction performance, we have implemented multiple mainstream GRN prediction algorithms including co-expression, Graphical Gaussian Models (GGMs), Context Likelihood of Relatedness (CLR), and parallelized versions of TIGRESS and GENIE3. Besides these existing algorithms, we also proposed a parallel Bayesian network learning algorithm, which can infer causal relationships (i.e., directionality of interaction) and scale up to several thousands of genes. Moreover, this web server also provides tools to allow integrative and comparative analysis between predicted GRNs obtained from different algorithms or experiments, as well as comparisons between legume species. The web site is available at http://legumegrn.noble.org.

## Introduction

In the post-genomics era, construction of gene regulatory networks (GRNs) and modelling gene interactions are important tasks in functional genomics and systems biology. The genome encodes thousands of genes whose products enable cell development and various cellular functions in response to diverse extracellular signals. Genes and gene products interact with each other to comprise a highly structured regulatory network. The accumulation of high-throughput gene expression data (such as, microarrays and RNA-seq data) provides great potential to uncover these complex gene regulatory networks that underlie biological functions, as those data provide snapshots of the transcriptome under many tested experimental conditions. For instance, legumes (*Fabaceae* or *Leguminosae*) constitute the third largest family of flowering plants and serve as an important source of food for humans and animals. Many legumes are capable of fixing atmospheric nitrogen through their symbiotic relationships with rhizobia bacteria. This symbiosis forms a major source of organic nitrogen fertilizer. Understanding this symbiosis is important for plant and microbial biology as well as for sustainable agriculture. In the past years, we have developed two important gene expression atlases for legume model species, i.e., *Medicago truncatula* [1], [2] and *Lotus japonicus* [3]. These two web sites have received wide attention from the legume community (e.g., the Medicago gene atlas has processed >100,000 analysis requests and has been cited over 200 times). The next step for these two gene atlases is to provide more complex analysis services in order to generate new knowledge about gene regulations and functions using GRN predictions.

To gain insight into these gene interactions, bioinformatics tools for GRN analysis are needed to generate hypotheses from high-throughput datasets. However, most of the current statistical or computational tools are difficult to access for most biologists. Although several web-based tools [4], [5], [6], [7], [8] have been developed to retrieve known or predicted gene-gene interactions based on existing knowledge, most of them are static databases and do not provide a dynamic GRN prediction function according to users’ requests. In other words, there is no way for end users to submit their own data or select specific datasets to perform personal GRN inferences. Two exceptions are GenePattern [9] and PredictiveNetworks [10]. However, PredictiveNetworks focuses exclusively on human datasets and GenePattern only provides GRN inference but no network query or visualization.

On other hand, numerous computational methods for GRN prediction have been recently proposed or applied. These methods include co-expression or relevance network (RNs) [11], graphical Gaussian modelling (GGM) [12], Boolean network [13], [14], differential equations [15], information theory [16], [17], Bayesian network (BN) [18], [19], regression models [20], among many others [21], [22]. However, two key problems still hinder their successful applications in practical GRN inferences. One is that the quality of network inference is not robust and stable [23], [24]. To illustrate this point, some studies [24] showed that half of algorithms only performed better than random guessing. A second problem is that some sophisticated models (such as, Bayesian networks) are time-consuming and infeasible for large datasets with several thousand genes and a large number of experiments.

To address these issues, we developed a flexible, open-source, web-based application and data service framework for GRN analysis using gene expression data (http://legumegrn.noble.org). In this web site, we integrated several commonly used GRN prediction algorithms including co-expression, GGMs [12], Context Likelihood of Relatedness (CLR) [16], GENIE3 [21], TIGRESS [20]. We parallelized GENIE3 and TIGRESS to make them feasible for large datasets. In addition, we also proposed a parallel version of the constraint-based BN learning algorithm called the PLPC algorithm, which is able to infer causal relationships or directionalities. To further improve prediction performance, our system is able to integrate prediction results from individual methods into a composite network to provide more accurate results. In addition to GRN prediction, we also implemented several features for GRN analysis such as GRN comparisons, GRN subnetwork query and GRN visualization. Although users are able to upload their own datasets, a special focus on legumes has been made by preloading into the web server all the Affymetrix GeneChip based gene expression data and annotation files publicly available from the three legume model species, *M. truncatula*, *L. japonicus* and *G. max*. Thus, the web site allows users to simply upload gene or probeset lists, and then select existing gene expression experiments (i.e., chips) and specific algorithms to perform GRN prediction in these three legume species. An additional feature allows users to finally identify conserved or divergent gene regulatory programs across these three species.

## Methods and Content

### Data Sets

To facilitate GRN analysis for legume species, we have collected all *M. truncatula*, *L. japonicus* and *G. max* Affymetrix microarray datasets from public EBI microarray database and from our collaborators. In total, we have collected 670 Medicago chips, 237 Lotus chips and 913 soybean chips. We then normalized all the raw data using Robust Multichip Averaging (RMA) through R and uploaded them into our database. To permit analysis of user-generated datasets or of data available from other species, we provided options for users to upload their own expression data in tab-delimited text format.

To check “batch effects” (the systematic error introduced by the different sources of data), we analysed transcriptomes of major plant tissues from experiments carried out at different locations for Medicago. From Principal Components Analysis (PCA), we observed that all the organs were well grouped in the PCA plot (Figure S1), which demonstrated that “batch effects” related to data sources are negligible in major organs of Medicago dataset. This analysis was not possible in Lotus and soybean due to the low redundancy of experiments between data sources. Moreover, to ensure users to compare relevant experiments, we provided correlation coefficients matrices between all experiment pairs for each species (the data are available at http://legumegrn.noble.org/cc.html).

### The GRN Inference Algorithms

To support custom GRN predictions, we implemented a multi-algorithm program that assists in the construction of gene networks for gene expression data. Multiple GRN prediction algorithms can complement each other and compensate for the limitations of a single GRN prediction approach to improve the prediction accuracy. Based on the literature and previous comparisons of GRNs [9], [25], we adopted GGM [12], co-expression [11], GENIE3 [21], TIGRESS [26] and CLR [16] as the major algorithms because of their good performances in each category of algorithms when testing over DREAM5 (Dialogue for Reverse Engineering Assessments and Methods) Network Challenge (http://www.the-dream-project.org/), a competition race in reverse engineering of GRNs [27]. We parallelized two GRN prediction algorithms, TIGRESS and GENIE3, to reduce their computation time for large data sets.

In addition, we also proposed a parallel constraint-based algorithm called PLPC (i.e., Parallel Low-order PC Algorithm), which is a parallelized version of our previous algorithm [25] based on a Bayesian network (BN) model. BNs are well suited for inferring gene networks because of their ability to model causal influence (cause-effect) between variables (i.e., genes). Most of BN learning algorithms are very time-consuming and hard to scale up to several thousand genes. Thus, we used parallel computing and restrained the highest order (means the size of conditioning set) in conditional independence (CI) tests to achieve feasibilities for large datasets; meanwhile, we also combined the idea used in the PC-stable algorithm [28] to improve performance. The details about this algorithm can be found in the File S1.

### GRN Integration

This integrative approach, also called ensemble analysis, has already been successfully applied in machine learning [29]. The basic idea of the ensemble analysis is that the combination of multiple models will obtain better predictive performance than any constituent individual algorithm. Previous studies [9], [30], [31] also demonstrated that ensemble analyses could be applied into GRN predictions and improve prediction accuracy.

Users can compare and integrate multiple networks predicted by different algorithms, and perform GRN integrative analyses using LegumeGRN. The web server is able to overlay multiple GRNs and construct a composite network, which allows users to investigate similarities and differences of multiple predicted structures. We use the adjacency matrix to combine and integrate multiple GRNs and generate the final combination result. In this procedure, we adopted an integrated score, where each edge is rescored using the average rank across all constituent inference methods. This integrative method, called Borda count election [32], was initially used for ranking candidates from a democratic election and has been successfully applied to GRN integrative analysis [9]. This method weights the confidence of each inferred interaction in this composite network, where each edge is rescored using the average rank across all *K* constituent inference methods (i.e., GRN prediction algorithms). The value is defined by a specific gene-gene connection (interaction) *I* predicted by the *i*th algorithm. Thus, the integrative value is:
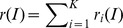
where *r_i_*(*I*) is the rank of the connection *I* predicted by the *i*th method. Intuitively, this value is the sum of predictions from individual approaches. Generally, this score will perform robustly across diverse datasets comparing to the score returned from each of the individual methods.

By combining the results of multiple algorithms, we observed an improvement of prediction accuracy due to the complementary advantages of each different individual algorithm (see case study 1).

### Across Species Comparison

Another feature available for legume species is the gene network comparison across different species (i.e., Medicago, Lotus and soybean). Many components of regulatory networks governing basic cellular functions are highly homologous in diverse species [11]. This comparative transcriptomics approach will enable detection of evolutionarily conserved GRNs. Comparison of integrated (multi-species) GRNs with single-species GRNs will also help place regulatory subnetworks (modules) into a phylogenetic context.

To compare GRNs in different species, we identified pairs of related genes (i.e., orthologous genes) between these three species. Each orthologous set was defined using a unique identifier, called a metagene ID. Identification of ortholog sets across multiple species was carried out using the BLAST algorithm and protein sequences. Protein sequences were downloaded for Lotus (ftp://ftp.kazusa.or.jp/pub/lotus/lotus_r2.5/), Medicago (ftp://ftp.jcvi.org/pub/data/m_truncatula/Mt3.5/Annotation/Mt3.5v5/) and soybean (ftp://ftp.jgi-psf.org/pub/compgen/phytozome/v9.0/Gmax/annotation/). We performed all-against-all BLASTP between each pair of protein sequences from each species using Reciprocal Best Hits (RBH). This method assumes that two genes residing in two different genomes are deemed orthologs if their protein products find each other as the best hit in the opposite genome. In this procedure, we used an E-value of 1×10^−6^ as the threshold and the option “*-F ‘m S’ –s T*” in BLASTP because these parameters have been demonstrated to better detect true functional orthologs [33]. We sorted the BLASTP hits from highest to lowest bit-score and if both the bit-scores and E-values were identical (i.e., more than one best hit), we considered them as multiple orthologs. In soybean, given its recent genome duplication, which led to multiple gene copies [34], we selected the best two hits from BLASTP results to include in the reciprocal blast analysis. All the probesets and gene IDs were mapped to metagene IDs. Thus, GRNs from different species can be compared with each other based on metagene IDs.

### Annotation Data

To facilitate GRN analysis, we implemented the web server with different functional annotations, such as KEGG annotation from GeneBins and GO terms [35], [36]. A module to identify significant enrichment in GO terms of each (sub)network using the chi-square test was developed and added to the web server. This feature may allow the identification of molecular functions or pathways in which most of the genes are involved. Additional gene annotations have been added, such as predicted transcription factor (TF) domains and tentative functional annotations for these three legume species. We also mapped the probeset IDs with related gene IDs obtained from IMGAG v.3.5 for Medicago, Lotus v.2.5 for Lotus and Soybase (http://soybase.org) for soybean. All these datasets were loaded into our databases to allow users to use Gene IDs or probeset IDs interchangeably as primary inputs.

### Software Implementation and GRN Visualization

LegumeGRN is a J2EE web application with Tomcat as the web server. The user interface was written in JSP and Servelet with a significant reliance on JavaScript language and JQuery libraries for front-end interactivity. AJAX was used for data retrieval in network visualization. Users can access the web page using any modern browser, including Microsoft Internet Explorer, Google Chrome, Apple Safari and Mozilla Firefox.

On the back-end, we have set up a multiple-host cluster and maintained a job scheduler using Oracle Grid Engineer to respond to user requests. We have implemented the GRN prediction algorithms in Matlab and R and deployed them into the cluster. JAVA was used to write the application for processing user requests, such as GRN prediction, subnetwork query, network comparison and integrative analysis. This application also accesses a MySQL database to manage the user’s personal analysis data, microarray datasets and annotation data.

On the client-side, we used the open-source Cytoscape Web [37] and AJAX to implement the visualization of GRNs on the web site.

### Utility

#### Personalized GRN analysis and workspace

To facilitate their use of the database, users may create an account in this server, which allows them to log in to submit new analysis tasks, and to access and manage their analysis results from previous sessions. Users can customize the datasets (gene/probeset lists, microarray chips), select suitable algorithms and tune the parameters as they wish, then submit GRN prediction requests. They will get an ID for every submitted analysis request. Afterwards, users can browse all requests in their own workspace and view job running status, query, compare GRNs and retrieve results using the job ID.

#### Primary input

**Figure 1:**
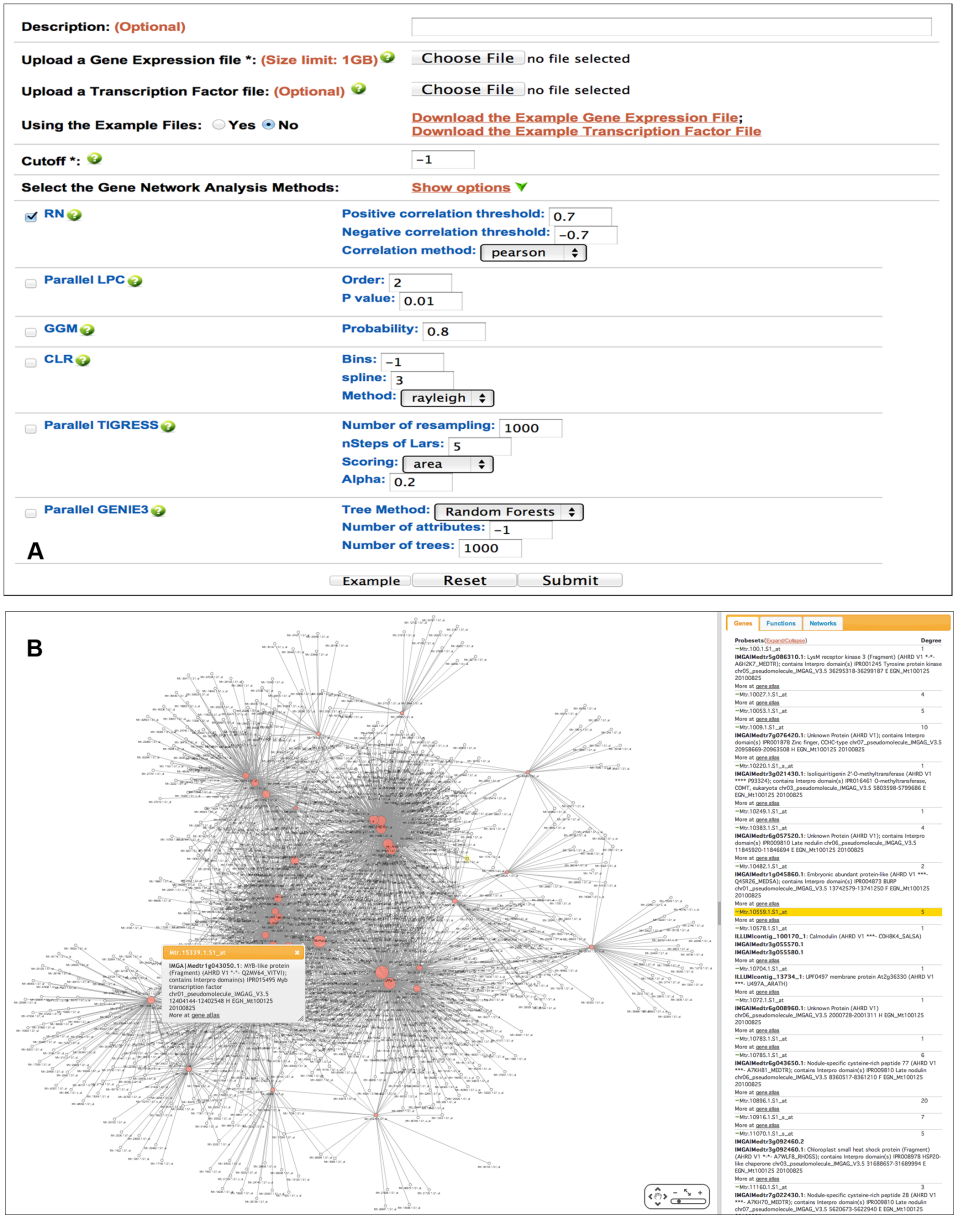
The snapshot of input and output web pages for GRN prediction. Figure 1A. A GRN prediction submission page. Figure 1B. A predicted co-expression network for 1,321 Medicago tissue-specific probesets according to the *Medicago truncatula* Gene Expression Atlas [1].

The primary inputs of legumeGRN are a gene expression data file and an optional transcription factor file. Then, users can select the prediction algorithms and tune the algorithm parameters to be used in the GRN construction (Figure 1A). A short description of different algorithms is provided, such as default parameters commonly used based in the published literature. For legume species, users simply need to upload their probeset or gene ID list and use checkboxes to select which transcriptomic samples they wish to use to build their GRNs.

#### Output

After calculation, GRN prediction results are saved into the LegumeGRN web server according to the users’ account. This feature allows users to store and keep track of their analyses and results. From the “analysis history” tab, users can retrieve all their prediction analyses with information related to analysis date, job title and description (optional), the transcriptomic dataset and parameters of predictive algorithms. At this step, users can download the network results and their related annotation information as tab-delimited text files or analyse them using an intuitive web-based GRN viewer to display the prediction results as graphical output. (Figure 1B).

The visualization module consists of a client applet with several features: display of genes as nodes and regulatory relationships as directed or undirected edges; zoom in/zoom out, and subset highlight. The connection strength and prediction approaches can be shown respectively according to line width and color. The annotation information (such as probeset ID, gene ID, tentative annotation, GO term) and links to related gene expression data are displayed by clicking on the node (i.e., gene model or probeset ID) in the viewer window, when information is available. Clicking on the edge displays the connection strength value returned by the corresponding GRN prediction algorithm and a link to show the gene expression profiles for the related gene pair. Users have also the ability to export the whole network as an image to either PNG or PDF file formats.

#### Subnetwork query

Global GRNs are usually too complex to be displayed and analysed in an individual web page. LegumeGRN allows users to generate a subnetwork from the predicted GRN. The subnetwork consists of the immediate connections of a specified gene list of interests or the first connections of transcription factors. Users have another option to select only the most important edges according to the confidence ranking, which is generated by the GRN prediction algorithm.

#### Network comparisons

Users are able to select multiple (sub-)networks using checkboxes and submit a GRN comparison task. A composite network is generated by comparison analysis and the GRN comparison within one species predicted by different algorithms or across several species can be visualized and downloaded from the web site.

For the GRN comparison within one species, each edge will be marked in different colors, with each color representing connections inferred by an individual prediction algorithm. The integrative score for each edge is calculated and listed in the text file.

Another comparison feature compares gene networks across the legume species available in legumeGRN (i.e., Medicago, Lotus and soybean). The composite network generated by this analysis includes metagene IDs and color-coded edges representing the network connections generated for each different species. The underlying related probeset IDs and gene IDs for each species can be displayed by clicking the node.

## Results

### Case Study 1

One of the DREAM challenges (i.e., DREAM5) is to reverse engineer gene regulatory networks from gene expression datasets (http://wiki.c2b2.columbia.edu/dream/index.php/D5c4). To validate the performance of our web site, we tested it using three datasets from the DREAM5 *in silico* network inference challenges. Two are experimental datasets obtained from microorganisms, *E. coli* and *S. cerevisiae*. The third one is based on an artificial network, which is a simulation dataset and derived from GeneNetWeaver [9]. We listed these three datasets in Table 1. Network predictions were evaluated on a subset of known interactions for each organism, or on the known network for the artificial case. Using LegumeGRN, evaluation of these datasets was performed to assess the performances of all six algorithms and their related integrative analysis for GRN combination. Although each algorithm has its own scoring measurements for the strength of edges (i.e., interactions), the ranked lists of interactions were compared against binary gold standard, performance was assessed by the area under the receiver operating characteristic (ROC) curve (AUROC) and the area under the precision vs. recall (PvsR) curve (AUPR). For a traditional ROC curve, recall (*N*_TP_/(*N*_TP_+*N*_FN_)) is plotted against 1-specificity (i.e., 1- *N*_TN_/(*N*_TN_+*N*_FP_)), and for a PvsR curve, precision (*N*_TP_/(*N*_TP_+*N*_FP_)) is plotted against the recall (*N*_TP_/(*N*_TP_+*N*_FN_)), where specificity, precision and recall are computed over a range of pruning thresholds, then the AUC values are obtained as the measurement scores, with higher scores indicating better performance.

**Table 1:** Three DREAM5 datasets used for performance evaluation in this study.

Dataset	|TF|	|Genes|	|Chips|
Artificial	195	1643	805
*E. coli*	334	4511	805
*S. cerevisiae*	333	5950	536

For the integrative analysis, we rescore each edge in the integrated networks using average rank across all three best inference algorithms.

We selected the top 100,000 edges returned from each approach according to their confidence rankings for AUC calculations. The AUPR and AUROC values of each approach are listed in Table 2. From Table 2, we observed that not a single method achieved best performance from all tests. However, the integrative GRN prediction results always achieved best or second best performance across all three datasets. It suggests that integrative analysis performed more stably than individual GRN prediction algorithms. The AUC scores are consistent with the test results reported in previous study [9].

**Table 2:** AUPR and AUROC scores for all six algorithms and one integrative analysis using three gold standard datasets from the DREAM5 challenge.

	AUPR			AUROC		
Algorithm	Artificial	*E.coli*	*S. cerevisiae*	Artificial	*E.coli*	*S. cerevisiae*
RN	0.1855	0.0129	0.0173	0.7516	0.4909	0.4998
GGM	0.0813	0.0872	0.0265	0.5883	0.5768	0.5269
Genie3	0.2837	0.0972	0.0206	0.8123	0.6200	0.5175
TIGRESS	0.3001	0.0608	0.0200	0.7602	0.5821	0.5158
CLR	0.2181	0.0804	0.0200	0.7558	0.5917	0.5129
PLPC	0.1339	0.0311	0.0179	0.5928	0.5142	0.5012
Integrative	0.2850	0.0999	0.0236	0.7910	0.6361	0.5359

Results from first best three algorithms were combined for the integrative GRN analysis. The best AUPR and AUROC results are underlined by solid lines and the second best ones are underlined by dotted lines in each column.

We further evaluated the accuracies of directionality prediction using the PLPC algorithm, which is the only one for predicting causal relationships. In Table 3, we listed the total edges, the true positives (TPs) and the true positives with correct directionalities predicted by PLPC for all three datasets. Here, we used *α* = 0.01 and *ord* = 8 as the input parameter. In all three cases, the directions for most of the edges can be predicted. Interestingly, ratios between TPs with correct directions and TPs are high. This indicates that PLPC has high chance to identify the correct regulation directionalities if edges are true positive. For the artificial network, we observed 30.1% precision ( = *N*_TP_/(*N*_TP_+*N*_FP_)) for the directed structure. The results showed that PLPC is a promising approach to discover causal directions when interactions are true. For the *E. coli* and *S. cerevisiae* datasets, directionality accuracy is still low in comparison to the artificial dataset, however performances are still reasonable when compared to other algorithms. Note that for these two experimental datasets, not all true regulatory relationships have been identified, thus the false positives may also represent true unknown relations.

**Table 3:** Prediction of directionality from PLPC for three DREAM5 datasets.

	Artificial		*E. coli*		*S. cerevisiae*	
	N. of Edges	TP	N. of Edges	TP	N. of Edges	TP
PLPC	1495(1493)	463(450)	1874(1871)	50(50)	1687(1679)	14(13)

For the PLPC algorithm, the numbers of predicted edges, predicted directed edges (in parenthesis), true positives and true edges with correct directionalities (listed in parenthesis) were listed.

### Case Study 2

In order to validate our web server on a legume dataset, we performed a co-expression analysis to identify putative target genes in *M. truncatula* of a well-studied transcription factor, *ABSCISIC ACID INSENSITIVE3* (*ABI3*). *ABI3* is known as a master regulator of seed maturation, which controls seed filling mechanisms and preparation for desiccation. This gene has been intensively studied in Arabidopsis and 78 of the *ABI3* regulons have been experimentally identified using chromatin-immunoprecipitation (ChIP-chip), array-based transcriptome, quantitative reverse-transcription-PCR (qRT-PCR) and transient promoter activation analyses [38]. These regulons encode proteins with known domains, which assigned them to various functions such as seed storage proteins, late embryogenesis abundant proteins, stress proteins, beta-glucosidases, or cytochrome P450s.

**Figure 2:**
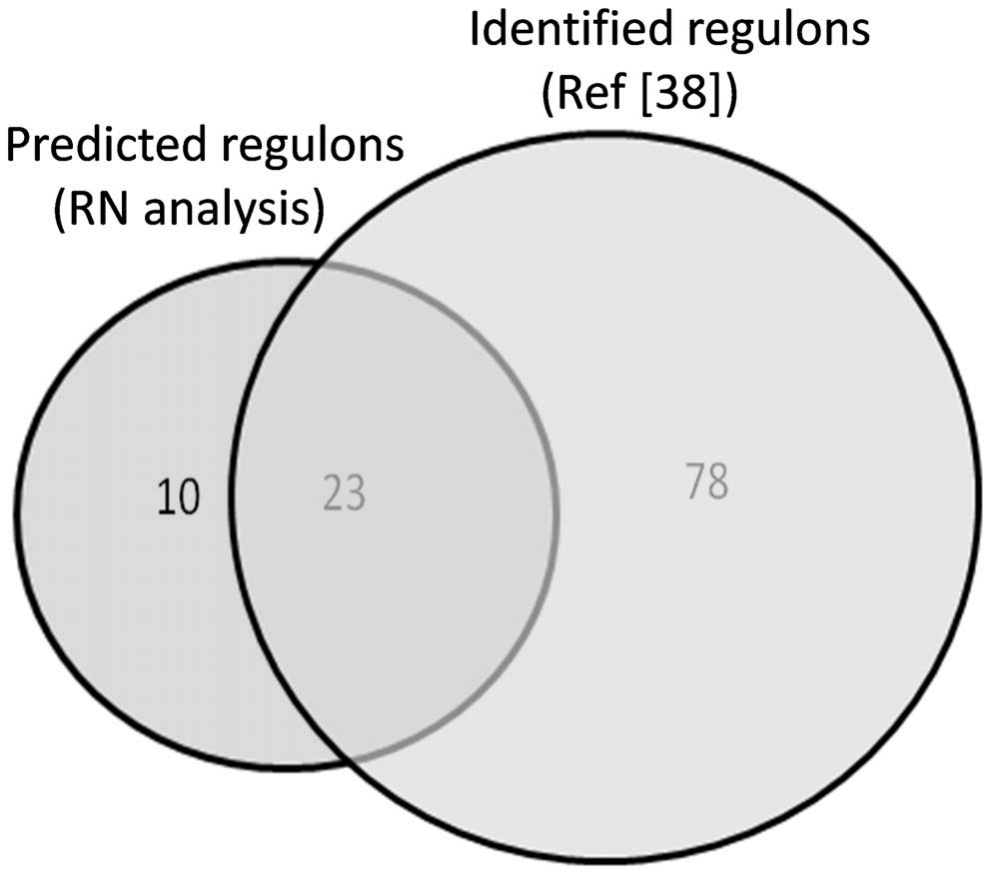
Venn diagram between identified ABI3 regulons [38] and predicted regulons according to LegumeGRN co-expression analysis (RN).

In this case study, we used the list of 1321 genes identified as tissue specific genes in *M. truncatula* according to the *Medicago truncatula* Gene Expression Atlas [1]. We submitted a gene network prediction using transcriptomic data from the seed developmental time series (i.e., 10, 12, 16, 20, 24, 36 days after pollination samples). We selected the co-expression (relevance) network as the analysis method using Pearson correlation coefficient with a positive threshold of 0.9 and negative threshold of -1 to identify putative positively regulated targets. We then submitted a subnetwork query with the probeset Mtr.44550.1.S1_at corresponding to *MtABI3*. From this subnetwork, we identified 44 putative regulons of *MtABI3* according to our algorithm parameters (Table S1). Using Affymetrix probeset tentative annotation and homology analysis with known sequences (i.e., BLASTX against IMGAG-v3.5, Swiss-Prot and TAIR9 datasets), we discovered protein domains and putative functions for 33 of them. Out of these 33 genes, 69.9% (i.e., 23/33) encode proteins, whose putative functions were shown to be regulated by *ABI3* in the plant model Arabidopsis [38] (Figure 2). Moreover, our predicted network identified two other transcription factors as regulons of *MtABI3*, *FUSCA3* (*FUS3*) and *DELAY OF GERMINATION1* (*DOG1*). These two genes have not yet been described as direct targets of *ABI3* but known to encode genes related to abscisic acid physiology and may act synergistically with *ABI3* to regulate an overlapping set of targets [38], [39], [40].

## Discussion

LegumeGRN provides one-stop services for biologists to predict GRNs using cutting-edge algorithms, who receive results in a user-friendly and intuitive visualization interface. Two case studies demonstrated that this web site is promising to identify the *in vivo* regulatory genetic networks.

Although our web tool is unique in allowing customization of data input, analysis algorithms and result visualization for the biologist with no bioinformatics training, there is still room to improve the performance of the GRN prediction in LegumeGRN. Even though GRN predictions on the artificial dataset (i.e., *in silico* dataset from DREAM5) performed well, predictions from microorganism or plant species datasets are less accurate presumably due to the more complex regulatory relationships. From the biological side, several solutions should overcome these problems in future, such as the increase of data resources, identification of *cis*-regulatory elements on promoter sequences and mutant gene expression data from plants impaired in gene expression. LegumeGRN web server will implement additional experimental data as soon as they are made publicly available to provide the latest information to biologists. From the computational side, we are interested in further improving GRN predictions using integrative analysis. In the machine learning field, algorithm diversity has been recognized as the key to the success of integrative analysis methods. Previous studies [41], [42] have showed that more diversity in result prediction resulted in higher efficiency after combining those results through an integrative analysis. Further efforts to define the best techniques to apply integrative analysis to GRN predictions might be beneficial.

## Supporting Information

Figure S1 **The PCA plot for the major organs (flower, leaf, nodule and root) for Medicago dataset.** We selected samples from same tissues but carried out by different labs for these four organs and removed control probesets, then, PCA on probesets were performed. The sample names used in PCA are: Flower, Flower 12 wk, Leaf, Leaf GUS-ox, Leaf IRG1 R108, Nod 14 dpi, Nod 14 dpi C, Root, Root A17 control, Root watered 4d.
(PDF)

Table S1 **Predicted regulons according to co-expression network.** Predicted regulons in seed development with related annotations, Pearson correlation coefficient values and functional annotations according to Arabidopsis homology.
(XLS)

File S1 **The description of the Parallel Low-order PC (PLPC) algorithm.** The formal pseudo codes of PLPC and simulation tests.
(PDF)
