# Supplementary figures and images for: LegumeGRN: A Gene Regulatory Network Prediction Server for Functional and Comparative Studies
Source: PLoS One. 2013 Jul 3;8(7):e67434. doi: 10.1371/journal.pone.0067434 (PMC3701055; doi:10.1371/journal.pone.0067434)

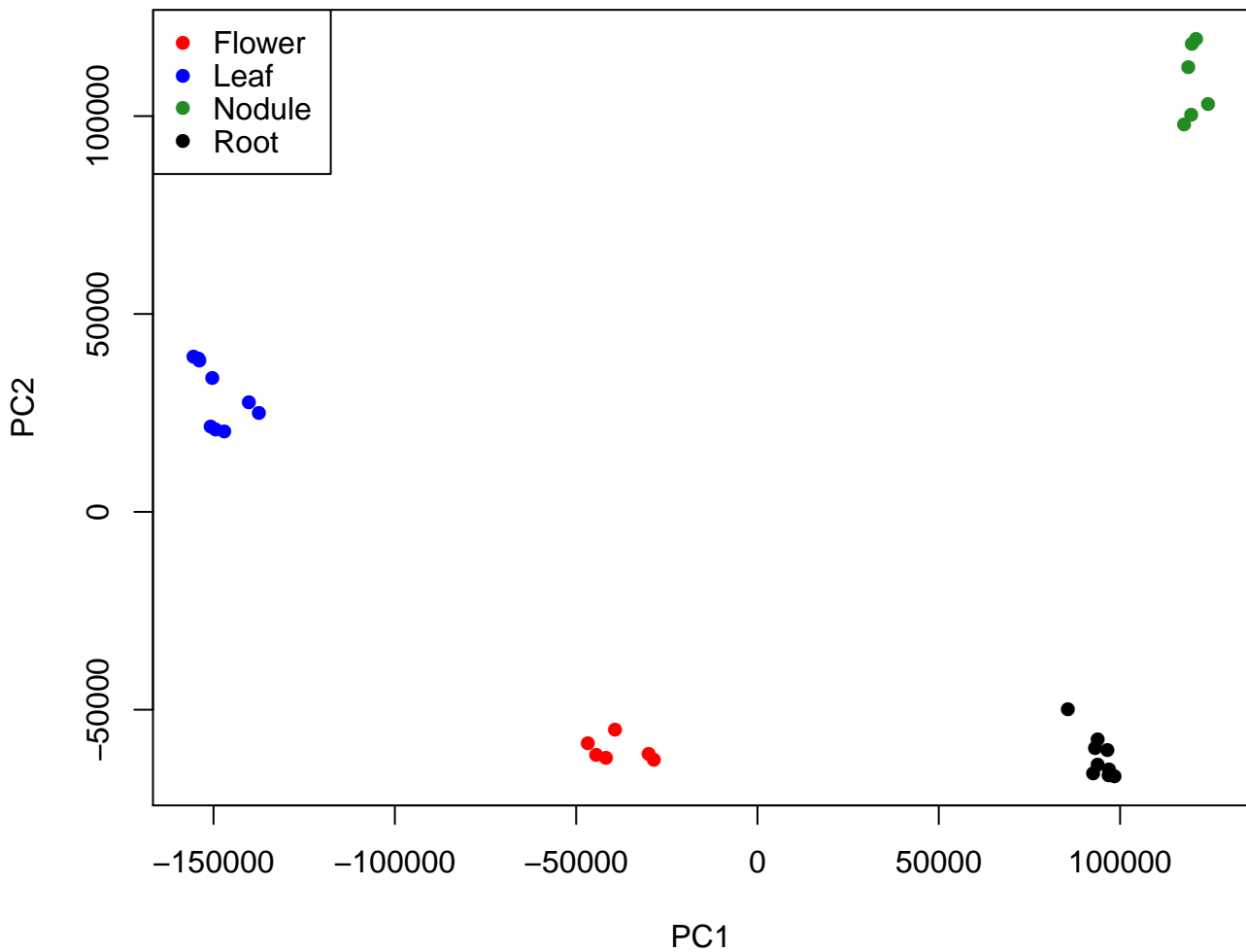

Supplement: Figure S1 — The PCA plot for the major organs (flower, leaf, nodule and root) for Medicago dataset. We selected samples from same tissues but carried out by different labs for these four organs and removed control probesets, then, PCA on probesets were performed. The sample names used in PCA are: Flower, Flower 12 wk, Leaf, Leaf GUS-ox, Leaf IRG1 R108, Nod 14 dpi, Nod 14 dpi C, Root, Root A17 control, Root watered 4d. (PDF) [file pone.0067434.s001.pdf]
