# Supplementary material for: LegumeGRN: A Gene Regulatory Network Prediction Server for Functional and Comparative Studies
Source: PLoS One. 2013 Jul 3;8(7):e67434. doi: 10.1371/journal.pone.0067434 (PMC3701055; doi:10.1371/journal.pone.0067434)
Supplement: File S1 — The description of the Parallel Low-order PC (PLPC) algorithm. The formal pseudo codes of PLPC and simulation tests. (PDF) [file pone.0067434.s003.pdf]

Supplementary Notes

1. Parallel Low-order PC Algorithm (PLPC)

1.1. Preliminaries

We proposed a parallel constraint-based algorithm for Bayesian network learning and included it into LegumeGRN. We consider using our previous developed LPC algorithm [1] to do parallelization for gene network predictions. An important reason for this is that this algorithm is the pairwise conditional independence (CI) tests used in this algorithm are easy to distribute into multicore systems and can therefore be carried out concurrently (i.e., effective parallelization can be achieved).

Before introducing this algorithm, we will give formal notations and definitions of Bayesian Networks (BNs) in this section. Most of information in this section can be found in most books on BNs, such as Ref. [2].

Let **V** denote a non-empty finite set of random variables. A Bayesian network (BN) for **V** is defined by a pair $<G,\mathbf{\Theta}>$.

The structural model is a directed acyclic graph (DAG) G=(**V**,**E**), in which nodes represent variables in **V** (in BN, variable and node can then be used interchangeably) and the set of edges **E** is all edges between nodes in **V**. We use the notation $X_i \rightarrow X_j$ if and only if there is a directed edge between two nodes X_i and X_j, and X_i — X_j if and only if there is an undirected edge between X_i and X_j. The *parents* of a node X_i (written **Parents**(G, X_i)) is the set of nodes that have directed edges to X_i. The *adjacency* set of a node X_i in graph G, denoted by **Adjacencies**(G, X_i), are all nodes that are directly connected to X_i by an edge. The elements of **Adjacencies**(G, X_i) are also called *neighbors* of X_i or *adjacent* to X_i. We call the set of edges connecting the k nodes a path from X_1 to X_k. X_j is called a *descendant* of X_i, and X_i is called an *ancestor* of X_j if there is a path from X_i to X_j, and X_j is called a non-descendant of X_i if X_j is not a descendant of X_i. For each node there is a probability distribution at that node given the state of its parents in G, denoted by $P(X_i|$**Parents**$(G, X_i))$. $\mathbf{\Theta}$ are parameters specifying all these probabilities. BNs follow the *Markov condition*, stating that given its parents each variable is independent of its *non-descendants*. Under the Markov assumption, each BN specifies a decomposition of the joint distribution over all distributions of the nodes, in a unique way: $P(\mathbf{V})=\Pi_{X_i\in\mathbf{V}} P(X_i|$**Parents**$(G, X_i))$.

It is necessary to give a brief description of the *conditional independence* (CI) relation. X_i and X_j are said to be conditionally independent given **S** (where $X_i \in \mathbf{V}$, $X_j \in \mathbf{V}$ and $\mathbf{S} \subseteq \mathbf{V}/\{X_i, X_j\}$) if $P(\mathbf{S}) \neq 0$ and one of the following holds: 1) $P(X_i \mid X_j, \mathbf{S}) = P(X_i|\mathbf{S})$ and $P(X_i|\mathbf{S}) \neq 0$, $P(X_j,\mathbf{S}) \neq 0$; 2) $P(X_i|\mathbf{S}) = 0$ or $P(X_j|\mathbf{S}) = 0$. This CI relation is denoted by $I(X_i, X_j|\mathbf{S})$. A CI relation is characterized by its *order*, which is simply the number of variables in the conditioning set **S**.

A criterion called *d-separation* captures exactly the CI relationships that are implied by the Markov condition. We say X_i and X_j are d-separated by a node set $\mathbf{S} \subseteq \mathbf{V}\backslash\{X_i, X_j\}$ in G if every path between X_i and X_j is blocked by **S**. A path between X_i and X_j is blocked by **S** if one of the following holds: (1) $W\in\mathbf{S}$ and W does not have converging arrows along the path between X_i and X_j, or (2) W has converging arrows along the path and neither W nor any of its descendants are in **S**. Here, we say a node W has *converging*

arrows along a path if two edges on the path point to W. A probability distribution $\mathbf{\Theta}$ on $\mathbf{V}$ is said to be *faithful* with respect to a graph G if conditional independencies of the distribution can be inferred from so-called d-separation in the graph G and vice-versa. More precisely, faithfulness of $\mathbf{\Theta}$ with respect to G means: for any X_i, $X_j \in \mathbf{V}$ with $X_i \neq X_j$ and any set $\mathbf{S} \subseteq \mathbf{V} \backslash \{X_i, X_j\}$, X_i and X_j are conditionally independent given $\mathbf{S}$ if and only if node X_i and node X_j are d-separated by the set $\mathbf{S}$. Based on the faithfulness assumption, we use information theoretic measures to detect conditional independence relation, then use the concept of d-separation to infer the structures of BNs.

The nodes X_i, X_j and X_k form a *v-structure* in a DAG G when $X_i \rightarrow X_j \leftarrow X_k$ is the subgraph of G induced by X_i, X_j and X_k. Two DAGs are equivalent if and when they represent the same d-separation statements. The equivalence class of a DAG G is the set of DAGs that are equivalent to G. Even given an infinite number of observations, we cannot distinguish among the different DAGs of an equivalence class. Using published results [3], we can characterize equivalent classes more precisely: two DAGs are equivalent if, and only if, they have the same skeleton and the same v-structures. The skeleton of any DAG is the undirected graph resulting from ignoring the directionality of every edge. A common tool for visualizing equivalence classes of DAGs is a partial directed acyclic graph (PDAG), which is a graph that contains both directed and undirected edges. There may be more than one PDAG that correspond to the same equivalence class because extra undirected edges can be oriented sometimes. Thus, completed PDAG (CPDAG) is proposed to represent an equivalence class uniquely [4]. The CPDAG corresponding to an equivalence class is the PDAG consisting of a direct edge for every compelled edge in the equivalence class, and an undirected edge for every reversible edge in the equivalence class. A directed edge $X_i \rightarrow X_j$ is compelled in G if for every DAG G' equivalent to G, $X_i \rightarrow X_j$ exists in G'. CPDAGs are also called maximally oriented graphs. Several orientation rules can be used to generate a CPDAG. The connections (edges) in a BN can be used to interpret causal relationships between nodes.

1.2. Algorithm and parallelization method

In this section, we present a new parallel algorithm for learning a PDAG from a database D with p nodes and n cases (called sample size). In our case, D represents a microarray dataset with p genes and n measurements/chips. The algorithm adopts similar procedures to those used in the classical PC-algorithm (named after its authors Peter and Clark) [5] but it requires only low-order CI tests and uses parallel computing, therefore, named a parallel low-order PC-algorithm, or PLPC. Thus, this algorithm can scale up to a dataset with several thousand nodes.

The formal pseudo-code of the PLPC-algorithm is presented in Supplemental Table 1. In this algorithm, it receives a dataset D, significance level ε, and maximal order *ord* of CI tests as input, and returns a PDAG as output. This PLPC-algorithm consists of two phases: CI tests and an orientation phase.

In the first phase, we use *ord* to limit the highest order in CI tests, i.e., only 0-*ord* CI tests are allowed in the algorithm. Generally, we specify *ord* as 2 or 3. Thus, only low-order CI tests are used in the first phase. There are two reasons to use low-order CI tests in first phase. Firstly, only performing low-order CI tests can restrain computational complexity. Although the PC-algorithm is able to apply for sparse graphs with several thousands of nodes, in worst case, the order may grow to p-2 and is still infeasible for large networks. Secondly, CI results are more reliable in low order tests. In the CI definition, to test $P(X|\mathbf{S})$, if there are many variables in the conditioning set $\mathbf{S}$, there may be very few examples in the data set that satisfies a particular value assignment for $\mathbf{S}$, and $P(X|\mathbf{S})$ may be inaccurate if there is noise in the

examples. Similar issues may occur for $P(X|Y,\mathbf{S})$. Thus, the high-order CI tests are hard to estimate if the sample size is small.

To guarantee that causal relationships inferred by this algorithm are correct, we need to perform extra tests in the orientation phase.

In the first phase, G is initiated as a fully connected undirected graph. Then, iterative CI tests are performed for each connected node pair given a node subset **S** taken from neighbor nodes of the connected node pairs. Under the DAG faithful assumption, correlations or non-correlations, direct or indirect correlations between node pairs can be distinguished by CI tests. In this procedure, we used $\boldsymbol{dep}_D(X_i, X_j|\mathbf{S})$ as a measure of the strength of the conditional dependence between X_i and X_j given **S** with respect to D. In order to decide if $I(X_i, X_j|\mathbf{S})$ is true or not, $\boldsymbol{dep}_D(X_i, X_j|\mathbf{S})$ runs a partial correlation coefficient calculation when D is continuous and then uses ε as the significance level. In our algorithm, the partial correlation coefficient calculation follows the method previously used in [5,6] and is described in the next section.

We applied the parallel computing in the first phase. The CI tests in the constraint-based algorithm are distributed into multiple available processors and run in parallel. The lines 9-18 are the loop of parallelization and share the variables G and **Sepset**.

Comparing to our previous LPC, another modification is that the PLPC algorithm stores all the adjacent nodes of each node into an array ($\mathbf{a}(G,X_i)$) before CI tests are performed (lines 4-6). This is the same idea used in the PC-stable algorithm [7], a recent variant of the original PC algorithm. The study [7] showed that the skeleton inferred from this new approach is more stable.

Typically, the CI tests take most of execution time. Therefore, the running time can be significantly reduced if CI tests are executed on several cores in parallel. Nowadays, multicore processors become the norm, thus, we can use the parallel loop (parfor) provided by the Matlab parallel toolbox to parallelize the lines 9-18 of Supplemental Table 1. To meet the requirements of this toolbox, we record G into two different data structure forms, one is the adjacency matrix (or a two-dimensional array) A as usual and the second one is one-dimensional array A_1 [0 to $p\times(p\text{-}1)/2\text{-}1$] containing the elements on upper triangle of this adjacency matrix. Each node pair can be represented by value 1 or 0 in this one-dimensional array, with indicating that these two nodes are connected or disconnected. The element $A_{i,j}(i<j)$ in the adjacency matrix can be converted as an index $(i\text{-}1)p+j\text{-}(i\text{-}1)i/2\text{-}i$ in the one-dimensional array A_1.

Using the adjacency matrix A, it will be easy to search adjacent nodes for each node and get related conditioning sets in line 10 of the PLPC algorithm. This adjacency matrix will not be updated during the parallel for loop (lines 9 to 18). Thus, it is equivalent to save the adjacent nodes for each node before CI tests as the PC-stable algorithm.

The node pairs are distributed equally among the available processors, each processor will carry out CI tests for assigned node pairs. The one-dimensional array A_1 is shared by multiple cores, and each element (entry) in this array is only updated by the processor which the corresponding node pair is running at. After each parallel loop ends, the values in A will be updated according to the newest values in A_1 which reflect the newest connection status of the network G. Thus, two data structures keep consistent again before enter next round for higher order CI tests.

In the second phase, the orientation rules are applied to orient the graph skeleton. The key point of the PLPC-algorithm is that the neighbor numbers for linked node pairs are checked before applying each orientation rule, i.e., min(|**Adjacencies**(G, X_i)\{X_j}|, |**Adjacencies**(G, X_j)\{X_i}|) ≤ *ord* given the node pair between X_i and X_j must be satisfied. This is a nontrivial step that must be completed before applying the orientation rules in the second phase. The goal of this step is to ensure that the orientation rules in the second phase are still correct when only 0-*ord* CI tests are performed in the first phase. First, the v-structures are determined (lines 23-25 of Supplemental Table 1) for triple nodes X_i, X_j and X_k, if X_i and X_j, and X_j and X_k are connected while X_i and X_k are not connected and $X_j \notin$**Sepset**(X_i, X_k), then we can infer directionality $X_i \rightarrow X_j \leftarrow X_k$ (any one of the three alternatives $X_i \rightarrow X_j \rightarrow X_k$, $X_i \leftarrow X_j \leftarrow X_k$ and $X_i \leftarrow X_j \rightarrow X_k$ will lead to $I(X_i, X_k|X_j)$ and $X_j \in$**Sepset**(X_i, X_k), and thus cause a contradiction). The other four orientation rules are given in lines 28-31 of the PLPC-algorithm (Supplemental Table 1). Those orientation rules (R1-R4) are repeatedly used to determine the directions of remaining undirected edges until no more edges can be oriented. The basic idea is to make sure that all other undirected edges can be oriented based on DAG assumption. Based on previous theoretical analysis in [4], the completeness and correctness of those orientation rules have been proved in [1] when only the low-order CI tests are used.

1.3. Partial correlation calculations

For conditional independence (CI) tests used in our algorithm, we followed the partial correlation calculation used in [5,6], that is, the sample partial correlation $\rho_{X,Y|\mathbf{S}}$, for any $X \in \mathbf{V}$, $Y \in \mathbf{V}$, $Z \in \mathbf{S}$ and $\mathbf{S} \subseteq \mathbf{V} \backslash \{X, Y\}$

$$\rho_{X,Y|\mathbf{S}} = \frac{\rho_{X,Y|\mathbf{S}\backslash Z} - \rho_{X,Z|\mathbf{S}\backslash Z}\,\rho_{Y,Z|\mathbf{S}\backslash Z}}{\sqrt{(1-\rho^2_{X,Y|\mathbf{S}\backslash Z})(1-\rho^2_{X,Y|\mathbf{S}\backslash Z})}} \tag{1}$$

The 0th-order partial correlation $\rho_{X,Y|\emptyset}$ is defined to be the regular Pearson correlation coefficient $\rho_{X,Y}$. Actually, the k-th order partial correlation (i.e., with $|\mathbf{S}|=k$) can be easily computed from three (k-1)th order partial correlations. Thus, the sample partial correlation $\rho_{X,Y|\mathbf{S}\backslash Z}$ can be calculated recursively by using (1). With the assumption that all involved variables are multivariate Gaussian, the partial correlation $\rho_{X,Y|\mathbf{S}}$ is zero if and only if X is conditional independent from Y given **S**.

For testing whether a partial correlation is zero or not, we apply Fisher's z-transformation of the partial correlation:

$$z(X,Y\,|\,\mathbf{S}) = \frac{1}{2}\log\left(\frac{1+\hat{\rho}_{X,Y|\mathbf{S}\backslash Z}}{1-\hat{\rho}_{X,Y|\mathbf{S}\backslash Z}}\right) \tag{2}$$

Classical decision theory yields the following rule when using significance level α. Reject the null-hypothesis $H_0(X,Y|\mathbf{S})$: $\rho_{X,Y|\mathbf{S}}=0$ against the two-sided alternative $H_A(X,Y|\mathbf{S})$: $\rho_{X,Y|\mathbf{S}} \neq 0$ if

$$\sqrt{m-|\mathbf{S}|-3}\,|z(X,Y\,|\,\mathbf{S})| > \Phi^{-1}(1-\alpha/2) \tag{3}$$

where $\Phi(\cdot)$ denotes the cumulative distribution function of a Gaussian distribution with zero mean and unit standard deviation and m is the sample size. Thus, the left-hand side of (3) is used as $\boldsymbol{dep}_{\mathrm{D}}(X,Y|\mathbf{S})$ and the right-hand side of (3) is used as the threshold ε in PLPC.

2. Comparing to the related algorithm

The PC-stable algorithm [7] is a recent modification of the classic PC algorithm. In this algorithm, the adjacency sets of all nodes are stored as $\mathbf{a}(X_i)$ in prior to CI tests under each order level l. The benefit of this scenario is that a wrong edge deletion will not affect following CI tests because the mistakenly removed edge at early stage will not change the adjacency nodes of all other nodes within a same order l, thus, avoid more errors from CI tests. The experiments in [7] have showed that PC-stable performed better than the original PC algorithm.

As mentioned in the last section, PLPC adopted the same idea of the PC-stable algorithm. The benefits are two folds. Firstly, in this way, PLPC can gain performance improvement as the PC-stable. The second one is addressing the technical constraint issue on the parallel computing. PLPC distributes the adjacency matrix on each processor before performing CI tests, thus, it does not need to communicate between the processors to modify the connections (edges) in the adjacency matrix (i.e., the two-dimensional array A) when the CI tests are executed on different processors.

The main difference between the PC-stable and PLPC algorithm is the latter one, which uses parallel computing and only carries out low order CI tests. Obviously, PLPC replaces full-order CI tests with lower-order CI tests so as to obtain a CPDAG that can be regarded as an approximation of the results returned by PC-stable while it can reduce the running time significantly. To check if PLPC still maintains the similar performance as PC-stable and time efficiency, we ran several tests over simulation datasets.

3. Simulation tests

3.1. Simulation data

We followed the same procedure used in [7] to generate a random DAG with a given number of vertices p and expected neighborhood size $E(N)$. The vertices are topologically ordered from low to high node number. We set $E(N)$=2. Only directions from a low order node to higher order nodes are allowed. According to this probability, we generated a related adjacency matrix A for 1 if have a connection in the lower triangle of the matrix and zeroes in the remaining entries. Next, we replaced the ones in A by independent realizations of a Uniform ([0.1,1]) random variable. A nonzero entry A_{ij} can be interpreted as an edge from X_j to X_i with "strength" A_{ij}, in the sense that X_1, …, X_p can be generated as follows: $X_1 = \varepsilon_1$ and $X_i = \sum_{r=1}^{i-1} A_{ir} X_r + \varepsilon_i$ for i=2, … , p, where ε_1, … , ε_p are mutual independent $\mathcal{N}(0,1)$ random variables. The variables X_1, …, X_p then have a multivariate Gaussian distribution with mean zero and covariance matrix $\sum(\mathbf{1} - A)^{-1}(\mathbf{1} - A)^{-T}$, where $\mathbf{1}$ is the $p \times p$ identity matrix. Using this method, we generated 10 datasets and each dataset includes 1000 variables and 50 samples.

We ran PC-stable and PLPC algorithms over 10 datasets using seven different threshold values, that is, $\alpha \in \{0.001, 0.002, 0.005, 0.01, 0.02, 0.05, 0.08\}$. We compared the execution time with core numbers varied from 1, 2, 4 and 8 respectively. Then, we used two measurements to do prediction accuracy comparisons.

3.2. Performance comparison results

First, we showed the average execution time used for the PLPC and PC-stable algorithms at α=0.01 for 10 runs. We ran all the tests on multiple-core AMD Opteron processor 6176 with 2.3 GHz. Supplemental Figure 1 showed the speedup of PLPC and PC-stable relative to the number of cores. This measurement is

used as a measure of the parallel efficiency and scalability. Both PLPC and PC-stable achieved a considerable speedup thanks to parallelization. We saw speedups of 1.9×, 3.5× and 5.6× for PLPC and speedups of 1.7 ×, 2.7× and 3.7× for PC-stable when utilizing 2 cores, 4 cores and 8 cores respectively. PLPC got more speedups than PC-stable using parallelization. The reason is the average numbers of CI tests assigned to each core in PLPC are less than PC-stable since we control the highest order are used for CI tests. Thus, the differences of CI tests' numbers on all processors are also less than PC-stable, and some processors take less idle waiting time in PLPC. Our PLPC is even 22%, 40% and 61% faster than PC-stable when using 2, 4 and 8 cores respectively because the computation time on higher-order CI tests are saved in PLPC.

Second, two metrics were used to compare prediction accuracies of these two algorithms for learning PDAG. One is the true positive (TP) number with fixed false positive (FP) numbers [8] (here 200 as the fixed FP number) as measures of the quality of network reconstruction and second is the Structural Hamming Distance (SHD) [9]. Both of these two metrics are used as the measurements of reconstruction quality. Briefly, the SHD counts the number of edge insertions, deletions, and flips required to convert the estimated PDAG into the correct representation (CPDAG) of the original DAG[10]. Thus, a large SHD indicates a poor fit, while a small SHD indicates a good fit. The test results using these two measurements were showed in Supplemental Figure 2. From these comparison results, we observed that two algorithms achieved similar performance when α is varied from 0.001 to 0.02. Only differences when α values are increased to 0.05 and 0.08. It is easy to understand that more weak connections are retained for high-order CI tests due to higher thresholds. We noticed that PC-stable got smaller SHDs than PLPC while PLPC got higher TP numbers for 0.05 and 0.08. It indicates that these two algorithms have very comparable performance for this simulation tests while running time has been reduced in PLPC.

Supplemental Table 1. Parallel Low-Order PC algorithm: PLPC

**Input**:	D:	a dataset containing p nodes in n cases
	ord:	the highest order can be performed in CI tests
	ε:	the threshold for CI tests
**Output:**	G:	the partial directed graph over n nodes

/* Phase 1: learn a graph skeleton */

Form a complete connected undirected graph G with n nodes;

Create the two-dimensional $p \times p$ arrays **Sepset**;

l:= 0;

repeat

repeat

for all nodes X_i in G

Let **a**(G, X_i)= **Adjacencies** (G, X_i)

end for

Select a new ordered pair of node X_i , X_j that are adjacent in G such that $|$**a**$(G, X_i)\backslash\{X_j\}| \geq l$;

Assign this node pair X_i , X_j into one available processor;

for each node pair X_i and X_j **in parallel do**

choose a new $\mathbf{S} \subseteq$ **a** $(G, X_i)\backslash\{X_j\}$ with $|\mathbf{S}|=l$;

if $\boldsymbol{dep_D}$ $(X_i, X_j|\mathbf{S}) < \varepsilon$ **then**

Delete edge X_i and X_j in G;

Save **S** in **Sepset**(X_i, X_j) and **Sepset**(X_j, X_i);

end if

If edge X_i and X_j is deleted or all $\mathbf{S} \subseteq$ **a**$(G, X_i)\backslash\{X_j\}$ with $|\mathbf{S}|=l$ have been chosen then

exit for

end if

end for

until all ordered pairs of adjacent variables X_i and X_j such that $|$**a**$(G, X_i)\backslash\{X_j\}|\geq l$ **and** $\mathbf{S} \subseteq$ **a**$(G, X_i)\backslash\{X_j\}$ with $|\mathbf{S}|= l$ have been tested for conditional independence

l:= l+1;

until there is no adjacent nodes X_i, X_j satisfying $|$ **a**$(G, X_i)\backslash\{X_j\}| \geq l$ **or** $l > ord$

/* Phase 2: Orientation */

for each triple of nodes X_i, X_j, X_k such that the pairs X_i, X_j and X_j, X_k are each adjacent in G but the pair X_i, X_k are not adjacent in G

if min(| **Adjacencies**$(G, X_i)\backslash\{X_j\}$|,| **Adjacencies**$(G, X_j)\backslash\{X_i\}$ |)≤ ord **and** min(| **Adjacencies**$(G, X_k)\backslash\{X_j\}$|,| **Adjacencies**$(G, X_j)\backslash\{X_k\}$|)≤ ord **and** $X_j \notin$ **Sepset**(X_i, X_k) **then**

orient X_i — X_j — X_k into $X_i \rightarrow X_j \leftarrow X_k$;

end if

next

repeat

R1 Orient X_j — X_k into $X_j \rightarrow X_k$ whenever there is arrow $X_i \rightarrow X_j$ such that X_i and X_k are nonadjacent **and** min(| **Adjacencies**$(G, X_j)\backslash\{X_k\}$|, **Adjacencies**$(G, X_k)\backslash\{X_j\}$|)≤ ord**;**

R2 Orient X_i — X_j into $X_i \rightarrow X_j$ whenever there is a chain $X_i \rightarrow X_k \rightarrow X_j$ **and** min(| **Adjacencies**$(G, X_i)\backslash\{X_j\}$|,| **Adjacencies**$(G, X_j)\backslash\{X_i\}$ |)≤ ord;

R3 Orient X_i — X_j into $X_i \rightarrow X_j$ whenever there are two chains X_i — $X_k \rightarrow X_j$ and X_i — $X_l \rightarrow X_j$ such that X_k and X_l are not adjacent **and** min(| **Adjacencies**$(G, X_i)\backslash\{X_k\}$|,| **Adjacencies**$(G, X_k)\backslash\{X_i\}$ |)≤ ord **and** min(| A**djacencies**$(G, X_i)\backslash\{X_l\}$|, | **Adjacencies**$(G, X_l)\backslash\{X_i\}$ |)≤ ord **and** min(|**Adjacencies**$(G, X_i)\backslash\{X_j\}$| ,| **Adjacencies**$(G, X_j)\backslash\{X_i\}$ |)≤ ord ;

R4 Orient X_i — X_j into $X_i \rightarrow X_j$ and orient X_l — X_j into $X_l \rightarrow X_j$ whenever there are two chains X_i —$X_k \rightarrow X_l$ and X_i — X_j — X_l — X_i such that X_k and X_j are nonadjacent **and** min(| **Adjacencies**$(G, X_i)\backslash\{X_j\}$|,| **Adjacencies**$(G, X_j)\backslash\{X_i\}$ |)≤ ord **and** min(| **Adjacencies**$(G, X_l)\backslash\{X_j\}$|,| **Adjacencies**$(G, X_j)\backslash\{X_l\}$|)≤ ord **and** min(| **Adjacencies**$(G, X_i)\backslash\{X_k\}$|,| **Adjacencies**$(G, X_k)\backslash\{X_i\}$ |)≤ ord **and** min(| **Adjacencies**$(G, X_i)\backslash\{X_l\}$|,| **Adjacencies**$(G, X_l)\backslash\{X_i\}$ |)≤ ord;

until no more edges in G can be oriented

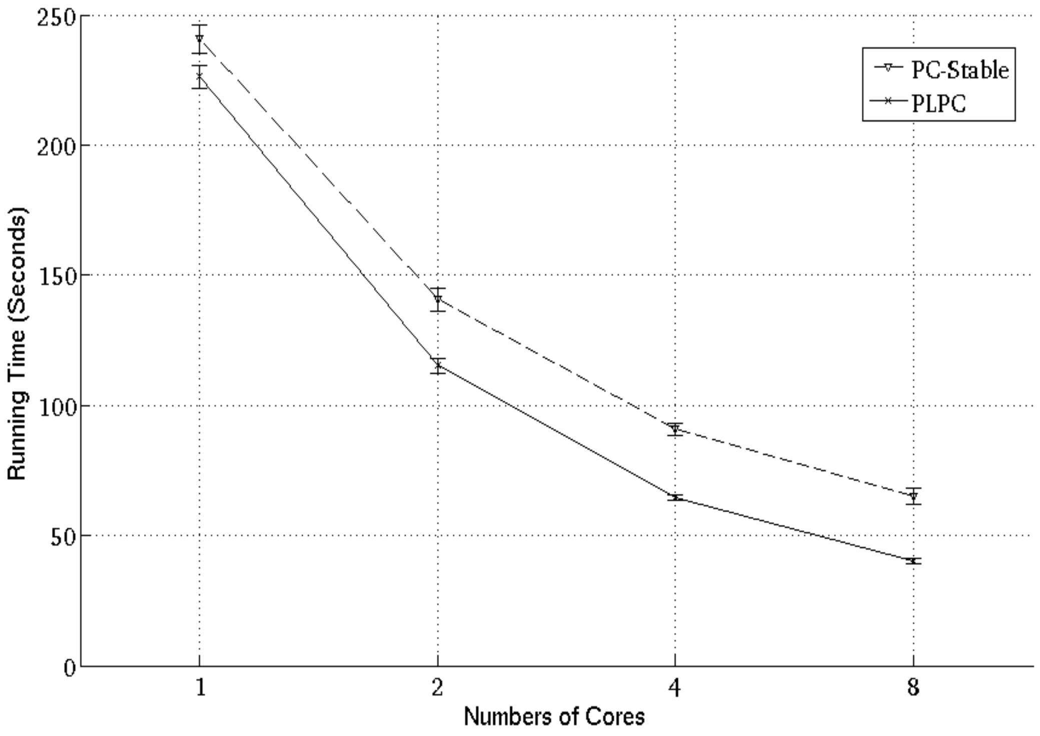

Supplemental Figure 1. Speedups of average running time over 10 datasets using the PLPC and PC-stable algorithms with increasing number of CPU cores. We used α=0.01 in both algorithms.

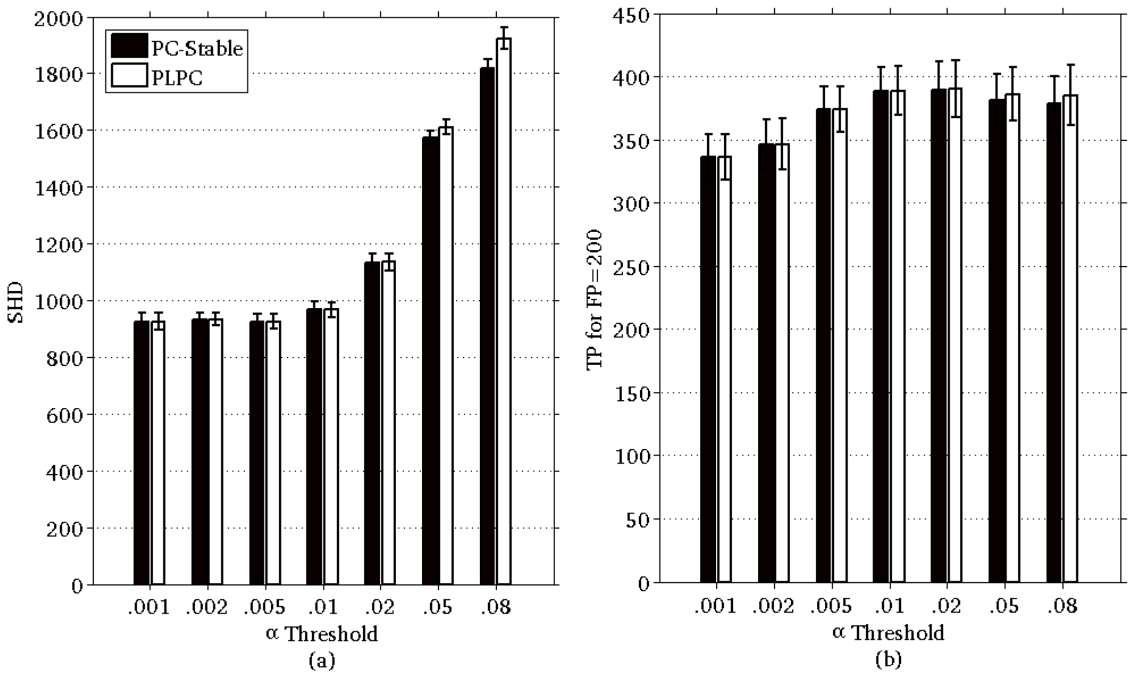

Supplemental Figure 2. The performance comparisons with different α thresholds for simulation tests. (a) Average SHD values for 10 runs. (b) Average true positives (TP) under 200 false positives (FP) for 10 runs.

References

1. Wang M, Augusto Benedito V, Xuechun Zhao P, Udvardi M (2010) Inferring large-scale gene regulatory networks using a low-order constraint-based algorithm. Mol Biosyst 6: 988-998.
2. Neapolitan R (2004) Learning bayesian networks: Prentice Hall Upper Saddle River, NJ.
3. Verma T, Pearl J. Equivalence and synthesis of causal models. In: Henrion M, Shachter R, Kanal L, Lemmer J, editors; 1990; Boston, MA, USA. Morgan Kaufmann, San Francisco, CA, USA. pp. 255-270.
4. Meek C. Causal inference and causal explanation with background Knowledge. In: Besnard P, Hanks S, editors; 1995; Montreal, QU, Canada. Morgan Kaufmann. pp. 403-418.
5. Spirtes P, Glymour C, Scheines R (2000) Causation, Prediction, and Search: MIT Press.
6. Kalisch M, Bühlmann P (2007) Estimating High-Dimensional Directed Acyclic Graphs with the PC-Algorithm. The Journal of Machine Learning Research 8: 613-636.
7. Colombo DM, Marloes H. (2012) A modification of the PC algorithm yielding order-independent skeletons. Preprint at <http://arxivorg/abs/12113295>.
8. Werhli AV, Grzegorczyk M, Husmeier D (2006) Comparative evaluation of reverse engineering gene regulatory networks with relevance networks, graphical gaussian models and bayesian networks. Bioinformatics 22: 2523-2531.
9. Tsamardinos I, Brown LE, Aliferis CF (2006) The max-min hill-climbing Bayesian network structure learning algorithm. Machine Learning 65: 31-78.
10. Chickering D (2002) Learning equivalence classes of Bayesian-network structures. The Journal of Machine Learning Research 2: 445-498.
